# Archaeal S-Layers: Overview and Current State of the Art

**DOI:** 10.3389/fmicb.2017.02597

**Published:** 2017-12-22

**Authors:** Thiago Rodrigues-Oliveira, Aline Belmok, Deborah Vasconcellos, Bernhard Schuster, Cynthia M. Kyaw

**Affiliations:** ^1^Department of Cell Biology, Institute of Biological Sciences, University of Brasília, Brasília, Brazil; ^2^Department of NanoBiotechnology, Institute for Synthetic Bioarchitectures, University of Natural Resources and Life Sciences, Vienna, Austria

**Keywords:** archaea, S-layer, cell envelope, protein glycosylation, protein structure

## Abstract

In contrast to bacteria, all archaea possess cell walls lacking peptidoglycan and a number of different cell envelope components have also been described. A paracrystalline protein surface layer, commonly referred to as S-layer, is present in nearly all archaea described to date. S-layers are composed of only one or two proteins and form different lattice structures. In this review, we summarize current understanding of archaeal S-layer proteins, discussing topics such as structure, lattice type distribution among archaeal phyla and glycosylation. The hexagonal lattice type is dominant within the phylum Euryarchaeota, while in the Crenarchaeota this feature is mainly associated with specific orders. S-layers exclusive to the Crenarchaeota have also been described, which are composed of two proteins. Information regarding S-layers in the remaining archaeal phyla is limited, mainly due to organism description through only culture-independent methods. Despite the numerous applied studies using bacterial S-layers, few reports have employed archaea as a study model. As such, archaeal S-layers represent an area for exploration in both basic and applied research.

## Introduction

The *Archaea* domain has characteristics in common with both the *Bacteria* and *Eukarya* domains, while at the same time exhibiting unique properties. When considering genes involved in replication, transcription, and translation molecular processes, this domain is more similar to *Eukarya*, whereas when analyzing genes involved in metabolic pathways it is more similar to *Bacteria* (Rivera et al., 1998). Characteristics particular to archaea include differences in cell membrane lipids and cell walls (Albers and Meyer, 2011), which vary in composition and, unlike bacteria, lack peptidoglycan (Kandler and König, 1978). In some archaea, polymers such as pseudomurein (Kandler and König, 1993) and methanochondroitin (Kreisl and Kandler, 1986) have been reported on cell envelopes, amongst other components. However, these polymers are mainly found in specific groups, with a protein surface layer, known as the S-layer, having been frequently detected in archaea (Sleytr, 1976; Sleytr et al., 2014). Indeed, this layer is present in certain bacteria and almost all archaea described to date (Albers and Meyer, 2011).

**FIGURE 1:**
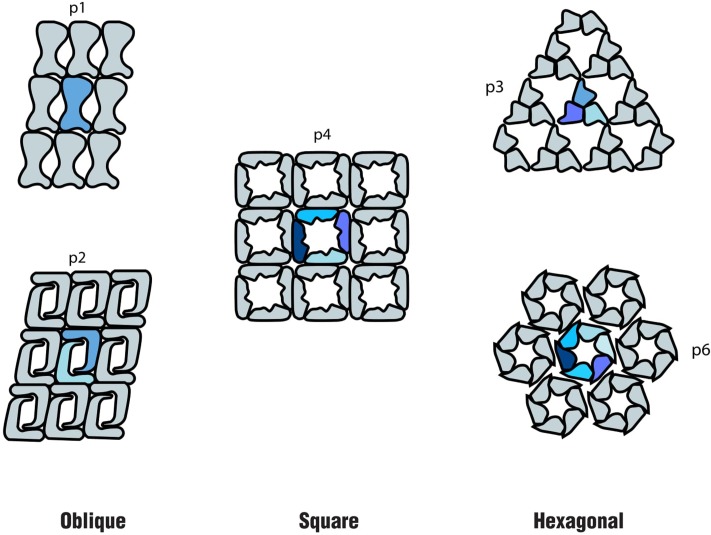
Schematics of the different S-layer lattice types, where the number of identical protein morphological units is highlighted: oblique (p1, p2), square (p4) and hexagonal (p3, p6) (based on the model proposed by Sleytr et al. (1999).

In both *Bacteria* and *Archaea*, S-layers are composed of only one or, in a few cases, two different (glyco) proteins. These are produced in large amounts within the cell and self-assemble into a paracrystalline surface layer (Sára and Sleytr, 2000; Sleytr et al., 2014). Depending on the organism, the S-layer lattice symmetry can consist of one (p1), two (p2), three (p3), four (p4), or six (p6) protein units, which results in regularly spaced pores (Sleytr et al., 2007; Pum and Sleytr, 2014) (**Figure 1**). As S-layers are monomolecular arrays of identical subunits, pores are identical in shape and size (Sára and Sleytr, 2000). Although the function of S-layers was initially not understood, they are now recognized to function as protective coats, molecular sieves, molecule and ion traps, as well as perform roles in surface recognition and cell shape maintenance (Sleytr et al., 2014).

Although studies on both archaeal and bacterial S-layer proteins have commonly reported an acidic isoelectric point (pI 3–5) (Sára and Sleytr, 2000), a much higher alkaline pI value (9.4–10.4) has been detected in lactobacilli (Hynönen and Palva, 2013). Other commonly reported S-layer protein features include the occurrence of 50–60% of hydrophobic amino acids and few sulfur-containing amino acid residues (Sára and Sleytr, 2000). Many known S-layer proteins can be *N*- or *O*-glycosylated, usually occurring on Asp and Ser or Thr residues (Messner and Sleytr, 1992; Jarrell et al., 2014; Schäffer and Messner, 2017). The glycan chains in bacterial S-layer proteins are generally composed of long repeating units of neutral hexoses, pentoses, heptoses, or amino sugars, while the glycan chains of archaeal S-layers tend to be shorter, with the exception of *Halobacterium salinarum* (Lechner and Wieland, 1989; Messner and Sleytr, 1991; Messner, 1997; Jarrell et al., 2014). Recently, evidence has been provided for the exposed glycan chains influencing the surface roughness of the cell on the nanometer scale and causing the formation of a lubricating hydration layer (Schuster and Sleytr, 2015). This presumable intrinsic feature of S-layer lattices may provide a self-cleaning surface structure (Herzog and Wirth, 2012; Schuster and Sleytr, 2015).

The S-layer is usually anchored to the microorganism surface and can be separated by the use of detergents or chemicals capable of breaking hydrogen bonds (Debabov, 2004). If such chemicals are removed, however, isolated units are capable of reassembly (Beveridge, 1994; Pum and Sleytr, 2014). Such a property has been extensively demonstrated to be suitable to different biotechnological applications (Debabov, 2004; Sleytr et al., 2007; Ilk et al., 2008; Pum et al., 2013; Schuster and Sleytr, 2014).

Although there have been several reviews on bacterial S-layer proteins and their biotechnological applications, detailed information on archaeal S-layers according to each archaeal phylum is limited. As such, the aim of this review is to provide a comprehensive and updated discussion of the state of the art of archaeal S-layer proteins among archaeal phyla, with focus on structure, glycosylation, lattice, assembly, and common features.

## Euryarchaeota

**FIGURE 2:**
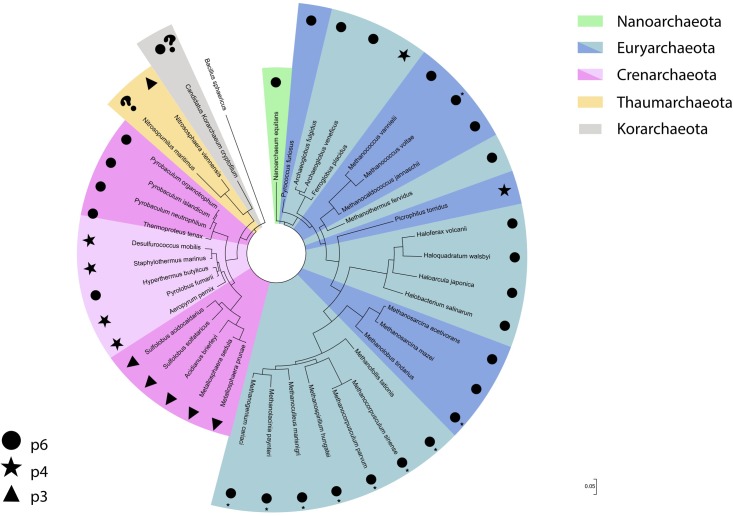
S-layer lattice type distribution across the different archaeal phyla displayed in a 16S rRNA gene based phylogenetic tree. The bacteria *Bacillus sphaericus* was used as an outgroup. ^∗^Described on the original study as hexagonal.

Although the Euryarchaeota phylum was formally proposed in 1990, along with the three domains of life system (Woese et al., 1990), euryarchaeotes were already being investigated decades prior to their reclassification. As such, reports regarding S-layer detection and characterization in organisms such as methanogens and halophiles were described prior to the proposal of the *Archaea* domain itself. These reports had a higher focus on cell morphology descriptions, while more recently proposed phyla have been detected mainly through culture independent methods. Thus, this has led to a myriad of information concerning the properties of S-layer proteins in these euryarchaeal groups when compared to more recently described organisms. It is also worth mentioning that the higher abundance of cultured isolates belonging to this phylum has greatly benefitted studies in this subject area. Interestingly, despite the high diversity of habitats and ecological lifestyles found in the Euryarchaeota, the S-layer is usually composed of only one protein, with the lattice type for most euryarchaeotal groups hexagonal (p6) (**Figure 2**).

### Methanogens

Methanogenic archaea have drawn attention from the scientific community ever since their detection in the early 1930s (Stephenson and Stickland, 1933), although at the time they were believed to be bacteria. As more studies on these organisms were performed, S-layers began to be frequently detected and some common characteristics have been observed. While information available on S-layer proteins for these organisms varies, for most a center-to-center spacing between 12 and 16 nm has been detected, though a smaller value has been observed for organisms such as *Methanogenium frigidum* (Franzmann et al., 1997). The cell envelope of *Methanospirillum hungatei* also displays unique features. While cells exhibit an S-layer similar to that observed in other methanogens (e.g., p6 lattice type; 15.1 nm lattice constant), they are further encased in protein sheaths which have an oblique (p2) symmetry (Firtel et al., 1993; Garcia et al., 2006).

Considering that the S-layer surrounds the entire cell as an important cell wall component, and often constituting the only cell envelope structure in archaea, it therefore represents a significant amount (10–15%) of the organism’s total protein content (Boot and Pouwels, 1996; Novotny et al., 2004; Sleytr et al., 2007, 2014). As such, continuous synthesis of the protein and a mechanism for controlling lattice growth are necessary. Although studies investigating this issue have been described in *Bacteria* (Chung et al., 2010), to our knowledge the only report using *Archaea* as a study model was performed with *Methanocorpusculum sinese* (Pum et al., 1991). For this methanogen, it was proposed that lattice faults such as disclinations and dislocations played an important role for incorporation of new lattice units during cell division. However, the lack of studies investigating this issue in other archaea leaves the comparability of this process among different groups open to speculation.

A number of studies have compared the S-layer of mesophilic and thermophilic methanogens. The S-layer of *Methanococcus vannielii, Methanococcus thermolithotrophicus*, and *Methanocaldococcus jannaschii* have shown comparable chemical and amino acid composition (Nußer and König, 1987; Akca et al., 2002), despite having different optimal growth temperatures (37, 65, and 85°C, respectively). There is no indication that these S-layer proteins are glycosylated when using PAS staining for detection (Akca et al., 2002). Although this could indicate that glycans are not required for the stability of the S-layer at higher temperatures, potential *N*-glycosylation sites for these methanococci have been detected when analyzing the protein’s amino acid sequence (Akca et al., 2002). Furthermore, the wide distribution of the archaeal glycosylation protein (agl) AglB indicates that *N*-glycosylation occurs in most organisms from this domain of life (Jarrell et al., 2014). While a role of glycosylation for S-layer proteins in methanogens had not been previously established, recent studies have suggested a higher glycosylation density in hyperthermophilic methanogens, which might play a role in survival in high temperature environments (Wildgruber et al., 1982; Zabel et al., 1984; Meyer and Albers, 2013; Jarrell et al., 2014). Although sharing certain similarities to the above mentioned S-layer proteins from other methanogens, the S-layer of *M. jannaschii*, an extreme thermophilic organism, displays a slight increase in the protein’s hydrophobicity, which has been suggested to contribute to lattice stability at higher temperatures (Jaenicke et al., 1985). Another distinct characteristic of the S-layer from this organism is that, when compared to mesophilic and thermophilic methanococci, the acidic amino acid Asp is predominant, in contrast to Ala. The basic amino acid Lys is also more frequent, with Cys and His residues observed which are not present in S-layers of other methanogens from this group. Interestingly, deduced secondary structure investigations indicated higher amounts of helical structures for the mesophilic *M. voltae* and *M. vannielii* S-layer proteins, while the thermophilic and extreme thermophilic *M. thermolithotrophicus* and *M. jannaschii* S-layer proteins exhibited more loops (Akca et al., 2002). Concerning S-layer protein secondary structure, a higher amount of β-sheet structures was observed in *Methanothermus fervidus* and *Methanothermus sociabilis* S-layer proteins (Brockl et al., 1991) when compared to mesophilic organisms (Baumeister et al., 1982; Bingle et al., 1985; Engelhardt et al., 1986). As β-structures interact both at intermolecular and intramolecular levels (Jaenicke, 1987), it has been suggested that the higher amounts of β-sheets observed might play a role in stabilizing the proteins and favor the crystalline lattice formation (Brockl et al., 1991). It is also worth mentioning that these proteins have a higher isoelectric point when compared to the S-layer proteins of other methanogens and an unusually high number of Ile, Tyr, Trp, Asn, and Cys residues.

The cell envelope of the mesophilic methanogen *Methanosarcina acetivorans* has been well documented. In freshwater medium, four-cell aggregates are formed and each cell is surrounded both by an S-layer and a methanochondroitin layer. In marine medium, by contrast, cells become isolated and are surrounded only by the S-layer (Sowers et al., 1984, 1993; Kreisl and Kandler, 1986; Baumeister and Lembcke, 1992). In both *M. acetivorans* and *Methanosarcina mazei*, tandemly duplicated S-layer protein DUF1608 domains have been characterized, which appear to be correlated to Methanosarcinaceae surface exposure. These proteins also seem to undergo *N*-glycosylation, with the sugars likely being α-D-linked mannose or α-D-glucose (Francoleon et al., 2009). Although S-layers are known to have unique properties and considerable biotechnological potential, there are very few detailed structural models for these proteins in the literature. The only report that investigated this issue in detail not only for methanogens but for any *Archaea*, was performed on the *M. acetivorans* S-layer protein (Arbing et al., 2012). In this study, the DUF1608 domain structure was determined and the model for the S-layer indicated that it is negatively charged and acts as a charge and size barrier that restricts molecule access to the cell periplasmic space. It was also possible to produce a working structural model for the 2D S-layer lattice, which improves our knowledge of the protein’s self assembly properties. Interestingly, a high content of β-sheets was detected and the β-sandwich folds were structurally homologous to eukaryotic virus protein envelopes, which has interesting implications for cell envelope structure evolution studies.

As already mentioned, S-layer proteins are frequently glycosylated, with the glycosylation process best understood in the methanogens *Methanothermus fervidus* (Hartmann and König, 1989) and *Methanococcus voltae* (Jarrell et al., 2014). In *M. voltae*, the *N*-glycosylation gene involved in the attachment of the final sugar to the glycan (*aglA*) and the gene involved in the transfer of the complete glycan (*aglB*) to the S-layer protein have been identified (Chaban et al., 2006). In *M. fervidus*, C-I-phosphate derivates of Man, Gal, GlcNAc, and GalNAC seem to serve as precursors for the biosynthesis of the S-layer glycoprotein. These derivates are converted to either guanosine diphosphate (GDP) or uridine diphosphate (UDP) activated forms, which are then connected to different types of UDP activated oligosaccharides. These can contain either exclusively neutral sugars or neutral and amino sugars. Interestingly, UDP activated oligosaccharides have been detected in the biosynthesis process of pseudomurein in other methanogens, a cell wall component which has also been detected concomitantly with an S-layer (Kandler and König, 1985; König et al., 1989; Albers and Meyer, 2011). Glc was detected in the activated oligosaccharides, which was suggested to have been formed by the epimerization of Man and, in a subsequent step, these Glc residues might also be 3-*O*-methylated. In later steps, the oligosaccharides are converted into dolichyl pyrophosphate activated forms and, because lipid activated precursors have also been found, it has been suggested that these might play a role in the glycoprotein and cell wall biosynthesis process (Hartmann and König, 1989). Indeed, it has been demonstrated that methanogens use dolichol monophosphate for glycan assembly, in contrast to Crenarchaeota, which use dolichol diphosphate (Taguchi et al., 2016).

Partial homology among S-layer protein genes of some methanogens has been reported (Yao et al., 1994). However, these sequences also differ to such an extent that a diversity of S-layer proteins in these organisms becomes evident. Given that surface structures possess a high evolution ratio due to direct contact with the environment, it is possible that this diversity mirrors the wide range of habitats in which methanogens occur (Kandler and König, 1985; Kandler, 1994; Yao et al., 1994).

The S-layer of all methanogenic groups studied to date exhibit a hexagonal lattice formation (**Figure 2**), similar center-to-center spacing distance, and a degree of homology between specific groups. However, when comparing gene or amino acid sequences in more phylogenetic distant organisms, no common denominator is apparent. Most S-layer proteins from these organisms also seem to be glycosylated and glycosylation density might be correlated to cell viability at high temperatures. Some remarkable features have been detected in these proteins when analyzing thermophilic methanogens, such as higher hydrophobicity and β-sheet structure amounts. These have been suggested to contribute to a more stable cell envelope in thermophilic conditions (Brockl et al., 1991).

### Halophiles

The earliest detection of halophilic archaea dates back to the end of the 19th century, with documentation of the appearance of pink stains in fish, meat and animal hides, all of which contained high amounts of salt for preservation purposes (Farlow, 1880; Clayton and Gibbs, 1927). As such staining was a problem for the salted cod industry (Kellerman, 1915; Browne, 1922), investigation of these pink stains was conducted, revealing the abundance of microorganisms now recognized as halophilic archaea (Litchfield, 1998). Since then, many studies have been performed on these organisms, with investigation in the halophilic archaeon *Halobacterium salinarum* S-layer enabling detailed description of the first prokaryotic glycoprotein (Mescher and Strominger, 1976), an important advance in this area of study. Like most euryarchaeotes, the S-layer lattice type in halophilic archaea is hexagonal and the center-to-center spacing distance value is similar to that detected in most methanogens. Other similarities between the S-layer proteins of the halophilic archaea described to date include a tendency for acidic amino acid composition, lacking cysteines, with comparable molecular weight and glycosylation. The S-layer is also the only cell wall component in these organisms, with the exception of halococci and certain strains of *Haloquadratum walsbyi* (Burns et al., 2007).

As mentioned earlier, the S-layer protein in *H. salinarum* drew significant attention from the scientific community as it was the first prokaryotic glycoprotein to be described in detail, with a carbohydrate content of 10–12%. Neutral hexoses, amino sugars and uronic acid have also been detected, which are linked to the protein both by *N*- and *O*-glycosylation processes (Mescher and Strominger, 1976). The predicted amino acid sequence of the protein indicates an N-terminal sequence of 34 hydrophobic amino acids which serve as a signal peptide and a 21 C-terminal amino acid residue stretch which likely serves as a membrane anchor domain. A high content of glycosylated threonine residues adjacent to this domain has also been detected (Lechner and Sumper, 1987). Interestingly, the S-layer of *H. salinarum* seems to be dependent on high salt concentrations for structural stability, considering that the hexagonal lattice pattern of the S-layer can be observed in membrane preparations at 5M NaCl but not at lower salt concentrations (Brown, 1964; Stoeckenius and Rowen, 1967; Steensland and Larsen, 1969). The reasons for this phenomenon, however, remain open to speculation, as there are no detailed models for folding in S-layer proteins from any halophilic archaea.

**FIGURE 3:**
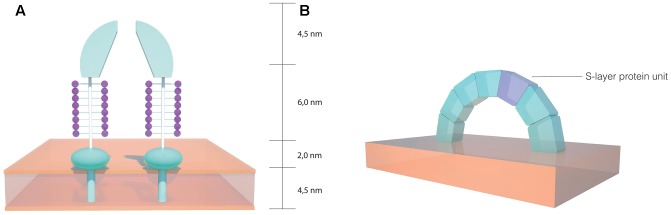
Schematics of the S-layer structure in halophilic archaea. **(A)** Dome shaped morphological complexes found on the *Haloferax volcanii* cell envelope (based on the model proposed by Kessel et al., 1988). **(B)** Arch shaped S-layer structure on the *Haloarcula japonica* cell surface (based on the model proposed by Horikoshi et al., 1993).

Two dimensional projection map studies have revealed a striking resemblance between the hexagonal arrangements of the S-layer morphological units from *H. salinarum* and the moderate halophilic archaeon *Haloferax volcanii* (Trachtenberg et al., 2000). *H. volcanii* has been a widely studied archaeon, serving as a model organism for archaea (Allers and Ngo, 2003; Hartman et al., 2010). In this organism, the S-layer protein shares many similarities to *H. salinarum*, with a high degree of homology between the two proteins. Nonetheless, a decrease has been detected near the N-terminal region, which indicates that there may be different architectures in the S-layer outermost sections. A signal peptide of the same length has been detected, as well as a putative membrane anchor domain near the C-terminal, preceded by threonine clusters which are likely *O*-glycosylated. These threonine clusters have been suggested to serve as a spacer between the membrane anchor domain and more distant parts of the protein. When compared to *H. salinarum*, fewer *N*-glycosylation sites occur on the *H. volcanii* S-layer protein and the carbohydrates involved are distinct (Sumper et al., 1990). As depicted in **Figure 3A**, three dimensional reconstructions of the *H. volcanii* cell envelope have shown that the S-layer is arranged as 12.5 nm high morphological complexes composed of a 4.5 nm dome-shaped domain with a narrow pore at the tip, followed by a 6.0 nm glycosylated spacer element (glycan chains represented in purple) and a small 2.0 nm globular domain next to the outer surface of the cell membrane (Kessel et al., 1988). This model indicates that the S-layer has a role as a selective molecular barrier for the cell, especially considering that it is the sole cell wall component on these organisms. A study using *H. volcanii* cell envelope preparations showed that the S-layer is dependent on NaCl and divalent cations for structural stability (Cohen et al., 1991), a property similar to that observed in *H. salinarum*. Taking into account the homology observed between the two proteins, it is likely that the salt and ionic conditions in the environment play a significant role in S-layer lattice structural stability in halophilic archaea.

**FIGURE 4:**
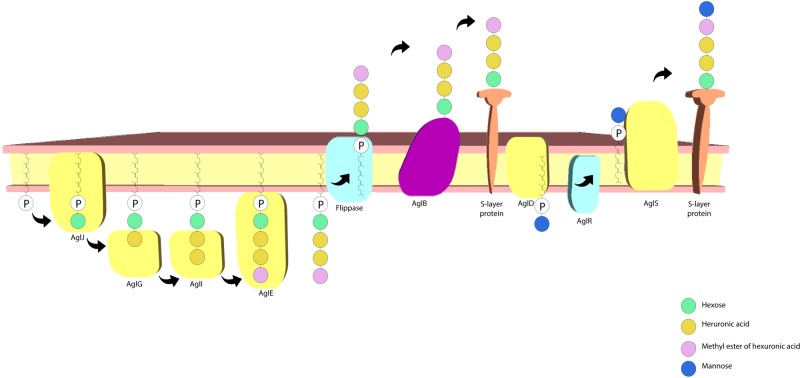
*Haloferax volcanii* S-layer protein *N*-glycosylation pathway. The archaeal glycosylation proteins AglJ, AglG, AglI, and AglE add a hexose, two hexuronic acid molecules and a methyl ester of hexuronic acid to a dolichol phosphate carrier, respectively. AglD adds a mannose to a separate dolichol phosphate carrier and AglB transfers the first four sugar residues to the S-layer protein. AglR flips the mannose bound dolichol phosphate carrier and AglS adds the final mannose to the protein (based on the model proposed by Jarrell et al., 2014).

The S-layer glycoprotein in *H. volcanii* has also been frequently used as a model for advancing understanding of post-translational modification in archaea. There is a sizable amount of data on this topic, with the protein *N*-glycosylation process having received considerable attention. A pentasaccharide comprising a hexose, a methyl ester of hexuronic acid, two hexuronic acids and a mannose molecule (Abu-Qarn et al., 2007; Guan et al., 2010; Magidovich et al., 2010) is linked to select Asn residues (Asn-13 and Asn-83). Many of the archaeal glycosylation proteins (Agl) involved in the process have also been identified, where AglJ, AglG, AglI, and AglE add the first four saccharide residues to one dolichol phosphate carrier (Guan et al., 2010) and AglD adds the final mannose to a different dolichol phosphate carrier (Abu-Qarn et al., 2008; Yurist-Doutsch et al., 2008; Kaminski et al., 2010). AglB transfers the four carrier-bound saccharides to the S-layer protein (Abu-Qarn et al., 2007). The final mannose residue is transferred from the other carrier to the glycoprotein, a process dependant on AglR and AglS. AglR flips the mannose linked dolichol phosphate and AglS transfers the final mannose to the glycoprotein (Guan et al., 2010; Calo et al., 2011; Cohen-Rosenzweig et al., 2012; Kaminski et al., 2012; Jarrell et al., 2014) (**Figure 4**). Interestingly, salt concentration in the medium affects the protein’s *N*-glycosylation process, with changes having been reported concerning both the glycans and the glycosylation sites in response to salinity changes (Abu-Qarn et al., 2007; Guan et al., 2012). The *N*-glycosylation of the Asn-498 residue of the protein constitutes a distinct process involving a tetrasaccharide which is dependent on lower salt concentrations (Kaminski et al., 2013). It has also been reported that *H. volcanii* cells have limited growth at high salt concentrations when deletion of the S-layer *N*-glycosylation pathway genes is performed (Abu-Qarn et al., 2007). This indicates that this process plays an important role in maintaining a stable cell envelope, ensuring survival in hypersaline environments.

Additional aspects of post-translational modifications have been reported for the *H. volcanii* S-layer protein. Studies have shown that the protein undergoes a magnesium ion dependent maturation step that occurs following translocation across the cell’s membrane (Eichler, 2001). This step appears to require lipid modifications on the protein by a derivate of mevalonic acid (Konrad and Eichler, 2002). Lipid modifications are also responsible for originating two *H. volcanii* S-layer protein populations: one is anchored to the membrane through a C-terminal transmembrane domain, while the other is lipid modified and associated with the membrane in a divalent cation dependent fashion (Sumper et al., 1990; Kandiba et al., 2013). Recently, it has been demonstrated that the protein’s C-terminus is removed by an archaeosortase (ArtA), with this enzyme acting on a conserved proline–glycine–phenylalanine (PGF) motif and the lipid modifications dependent on both ArtA and PGF (Abdul Halim et al., 2015). These studies have together advanced our understanding of the nature of post-translational modifications in archaea, a topic where information is relatively scarce.

The S-layer proteins of other archaeal halophiles have received less attention than those in both *H. salinarum* and *H. volcanii*. The triangularly shaped *Haloarcula japonica* cell envelope consists of an S-layer glycoprotein with similarities to those present in other halophiles, such as the presence of a 34 hydrophobic amino acid stretch that likely acts as a signal peptide, as well as a probable membrane anchor domain near the C-terminal portion. The size and the amino acid composition of the protein are also similar, although there are fewer potential *N*-glycosylation sites and their location is also different to that detected in *H. salinarum* and *H. volcanii* (Wakai et al., 1997). It has been proposed that the *H. japonica* S-layer proteins are arranged in an arch-like structure (**Figure 3B**) on the cell surface and it has been reported that magnesium ions are required for S-layer structural stability, with the release of less than 5% of the proteins from the cell membrane causing drastic morphological changes (Horikoshi et al., 1993). The square shaped *Haloquadratum walsbyi* has also been reported to have an S-layer surrounding the membrane, although some strains exhibited a more complex three-layered structure on the cell wall (Burns et al., 2007). Based on genomic studies, the S-layer of *H. walsbyi* has been suggested to have similarities to other halophiles, particularly *H. japonica* (Dyall-Smith et al., 2011). Considering that both species are known for their distinct geometric shapes, it is likely that the S-layer plays an important role in maintaining cell shape and morphology in these organisms.

When compared to methanogens, the halophilic S-layer proteins share more common characteristics, with similarities in features such as gene and amino acid sequences, hydrophobicity, signal peptides, and the presence of a membrane anchor domain close to the C-terminal portion. Salinity seems to play an important role in cell envelope and lattice stability. Furthermore, the role of glycosylation in S-layer proteins from halophiles is closely related to cell viability in hypersaline environments.

### Other Euryarchaeotes

There is noticeably less information in the literature concerning the S-layers of the remaining euryarchaeal groups when compared to methanogens and halophiles. However, it is worth pointing out that the *Ferroglobus placidus* S-layer exhibits a lattice type of orthogonal symmetry (p2 or p4) and a lattice constant of 23 nm, two characteristics that differ from that reported in other euryarchaeotes (Hafenbradl et al., 1996). Within the same order as *F. placidus*, the Archaeoglobales, *Archaeoglobus veneficus* has also been shown to exhibit a higher value for the S-layer lattice constant (19 nm), although the lattice type is hexagonal (Huber et al., 1997). The S-layer of the hyperacidophilic *Picrophilus oshimae* and *Picrophilus torridus* also differ from methanogens and halophiles in terms of lattice type symmetry, with a tetragonal (p4) lattice with a lattice constant value of 20 nm reported for these hyperacidophilic archaea (Schleper et al., 1995, 1996).

A double S-layer has been reported as a common feature in many species belonging to the order Thermococcales (Miroshnichenko et al., 1998; Kostyukova et al., 1999; Atomi et al., 2004; Gorlas et al., 2014). Electron microscopy analysis of the cell envelope of *Thermococcus stetteri* revealed a double layer of regularly packed glycoproteins, each 5 nm in width, separated by a weakly contrasted area of 10–12 nm (Gongadze et al., 1993). Curiously, a further proteinaceous layer, with similar morphology and size, was found to be attached to the cell membrane on the cytoplasmatic side. A role in stabilization under the extreme conditions has been proposed for this inner layer in these organisms (Gongadze et al., 1993). Production of a large number of membrane vesicles (MVs) and nanotubes coated by S-layer has also been reported in some *Thermococcus* species (Soler et al., 2008; Marguet et al., 2013; Gorlas et al., 2014). Many *Thermococcus* MVs were shown to carry DNA and, therefore, it has been suggested as a mechanism in genetic transfer between cells at high temperatures (Soler et al., 2008; Gorlas et al., 2015). Recently, a study performed by Gorlas et al. (2015) detected the production of sulfur vesicles (SVs) incased by S-layers in thermococci grown in media containing elemental sulfur. Although cryo-electron micrographs revealed a clear S-layer structure covering SVs during the budding process from the cell, cytoplasmatic membrane could not be detected in these vesicles. Since SVs could not be purified in vesicle preparations and were rarely observed in the free form, it has been proposed that the direct contact between sulfur and S-layers could lead to SV instability and vesicle disruption shortly after release (Gorlas et al., 2015).

Although there are common features present in the S-layer of organisms from the phylum Euryarchaeota, marked differences in structure, environmental conditions for lattice stability and glycosylation can be observed. It is worth pointing out that there is a lot more information on the methanogenic and halophilic hexagonal S-layers, which can be related to the higher number of cultured isolates belonging to these groups. However, as discussed earlier, there are fewer studied euryarchaeal groups that exhibit non-hexagonal lattice type symmetries on their S-layer and it is possible that this might become a more common occurrence with the isolation in pure culture of novel euryarchaeotal organisms.

## Crenarchaeota

Together with the Euryarchaeota, the Crenarchaeota phylum was also described in the *Archaea* domain proposition (Woese et al., 1990). This phylum is composed exclusively of thermophilic and hypertermophilic organisms. The first described members of this group were isolated from natural acidic thermal habitats around 45 years ago (Brock et al., 1972). In the following years, many crenarchaeotes from a variety of thermal environments have been isolated and further characterized (Zillig et al., 1981; Stetter, 1982; Fiala et al., 1986; Baumeister et al., 1991), making it, after Euryarchaeota, the second archaeal lineage with the most cultured representatives. For this reason, the cell wall composition of some crenarchaeal species has been described in detail and, in some cases, their S-layers thoroughly investigated.

Based on morphological, physiological and molecular characteristics, the orders Sulfolobales, Desulfurococcales, and Thermoproteales were described within the crenarchaeotal branch (Huber, 2006). Interestingly, with the notable exceptions of *Thermosphaera aggregans* (Huber et al., 1998) and species of the *Ignicoccus* spp. genus (Huber et al., 2012), both belonging to Desulfurococcales, all known organisms of these three orders have an S-layer as their sole cell wall component anchored directly to the cytoplasmatic membrane, enclosing a quasi-periplasmic space. Recently, the isolation of new representatives with distinctive nucleotide signatures in their 16S rRNA genes and phenotypic properties led to the proposal of two new orders within the Crenarchaeota: Fervidicoccales and Acidilobales (Prokofeva et al., 2009; Perevalova et al., 2010). However, although initial morphological descriptions from the few cultured members affiliated to these orders suggested the presence of S-layers (Boyd et al., 2007; Prokofeva et al., 2009; Perevalova et al., 2010), detailed analyses regarding their cell envelope structure and composition have yet to be conducted.

Among the crenarchaeotes, currently available data suggests that the S-layer structural features correlate with the organism’s phylogeny, with members of closely related taxa usually sharing similar S-layer characteristics (Rachel, 2010). For this reason, S-layers of the main Crenarchaeota orders (Sulfolobales, Desulfurococcales, and Thermoproteales) will be discussed separately in the following sections.

### Sulfolobales

The order Sulfolobales comprises thermoacidophilic organisms, with optimal growth temperatures between 65 and 90°C and pH around 2 (Huber and Prangishvili, 2006). Although initial electron microscopy of the species *Sulfolobus acidocaldarius*, in the early 1980s, assigned a p6 lattice arrangement for its surface layer (Taylor et al., 1982; Deatherage et al., 1983), further studies employing imaging processing strategies revealed a p3 symmetry, with 60° rotations between the trimeric motifs and the presence of twin boundaries (Lembckbe et al., 1991). A p3 lattice symmetry was also later observed in other species of Sulfolobales (**Figure 2**), such as *Sulfolobus solfataricus* (Veith et al., 2009), *Sulfolobus shibatae* (Lembckre et al., 1993), *Metallosphaera sedula* (Fuchs et al., 1995), *Metallosphaera prunae* (Fuchs et al., 1995; Veith et al., 2009) and *Acidianus brierleyi* (Baumeister et al., 1991). Indeed, this appears to be a common feature to all organisms described in this crenarchaeal order. Other S-layer structural characteristics seem to be very similar among all the Sulfolobales, with a center-to-center spacing of around 20 nm and a periplasmic width of about 25 nm being reported in all currently described species (Rachel, 2010).

Crystallographic approaches and imaging analyses conducted in the last few decades have provided important insights into S-layer conformation and indicate a very similar structure among Sulfolobales species (Inatomi et al., 1983; Baumeister et al., 1991; Lembckre et al., 1993; Veith et al., 2009). Three dimensional reconstructions revealed a smooth external surface layer and a rough internal surface exhibiting a dome shape cavity centered in a three-fold axis, with protruding filaments (Baumeister et al., 1991; Lembckre et al., 1993). Further biochemical studies (Grogan, 1989, 1996) revealed the S-layers to be composed of two dissimilar highly glycosylated proteins. The latter, now known as SlaA and SlaB (Veith et al., 2009), are non-covalently associated and have distinctive structural roles: SlaA glycoproteins form the highly ordered outer sheath and SlaB glycoproteins form stalks that anchor it to the cell membrane, constituting the observed filamentous protrusions.

Computational predictions of SlaB proteins performed by Veith et al. (2009) suggested that this protein is composed of two to three beta sandwich domains and a coiled coil region, which extends straight from the cell surface and forms a 20 nm stalk comprised of three copies of the protein, with the hydrophobic core inside. SlaB trimers seem to be anchored to the membrane via a C-terminal transmembranic helix, a feature conserved among the Crenarchaeota. SlaA predictions indicate the presence of a dimeric molecule building the sacculus at a ratio of three dimers to one triangular pore. Based on these results, a hypothetical model for Sulfolobales S-layers has been proposed (Veith et al., 2009). Although possible explanations on how SlaA and SlaB are connected to each other have been raised, the specific mechanisms by which these proteins interact remain unclear. Interestingly, despite the typically rare detection of Cys residues in bacterial S-layer proteins (Sára and Sleytr, 2000), these have been detected in SlaA and provide thiol groups that have been used for magnetic gold (Au) nanoparticle production (Selenska-Pobell et al., 2011).

Furthermore, studies on the bindosome assembly system (Bas) and its role in *S. acidocaldarius* indicated that sugar binding proteins are present in high molecular mass complexes functionally associated to the S-layer (Zolghadr et al., 2011). Deletion of Bas system components led to S-layer lattice disturbances, suggesting that bindosomes are a structural component of the *S. acidocaldarius* cell envelope and contribute to its shape.

It has been suggested that the *S. acidocaldarius* S-layer protein plays a role in the anchoring process of the archaellum (archaeal flagella) (Banerjee et al., 2015). FlaF is one of the seven proteins of the archaellum and binds to the S-layer protein, with this interaction occurring through a domain located on the pseudoperiplasm. Thus, FlaF may be responsible for anchoring the rotating archaellum to the *S. acidocaldarius* cell envelope. Curiously, this study also revealed that FlaF is structurally similar to the *Geobacillus stearothermophilus* SbsB S-layer protein.

Glycosylation of the *S. acidocaldarius* S-layer protein has been investigated (Peyfoon et al., 2010; Meyer and Albers, 2013) and the mature protein has 31 predicted *N*-glycosylation sites, with one third being concentrated on the C-terminal domain, constituting a remarkable glycosylation density. Interestingly, this high glycosylation density has also been detected in amino acid sequences from all Sulfolobales, suggesting that this might be an adaptation to high temperature and acidic environments (Meyer and Albers, 2013). Each site is modified with heterogeneous glycan families that are linked via chitobiose core disaccharides, a feature common in *N*-glycosylation processes observed in the *Eukarya* domain (Peyfoon et al., 2010).

Molecular analyses have shown that the *slaA* and *slaB* genes lie adjacent in chromosomes and are constitutively transcribed as bicistronic operons, a feature conserved in the Sulfolobales (Veith et al., 2009). Although it has been observed that cell transcriptional levels of *slaA* are much higher than *slaB*, multiple sequence alignment of the intergenic regions suggests a conservation of the transcriptional and translational regulatory pathways by which Sulfolobales adjust the expression of S-layer genes (Veith et al., 2009). Additionally, while no SlaA homolog has been found in organisms other than the Sulfolobales, SlaB seems to be distantly similar to the S-layers of other Crenarchaeota, such as the *Staphylothermus marinus* tetrabrachion (Veith et al., 2009).

### Desulfurococcales

All the Desulfurococcales members described to date are hyperthermophiles, with optimal growth temperature between 85 and 106°C. Unlike the Sulfolobales, however, they are mostly neutrophilic and with many unable to use sulfur components for energy production (Huber and Stetter, 2006). Based on phylogenetic and physiological differences, there are currently two families assigned to this order: Desulfurococcaceae and Pyrodictiaceae (Huber and Stetter, 2015a).

**FIGURE 5:**
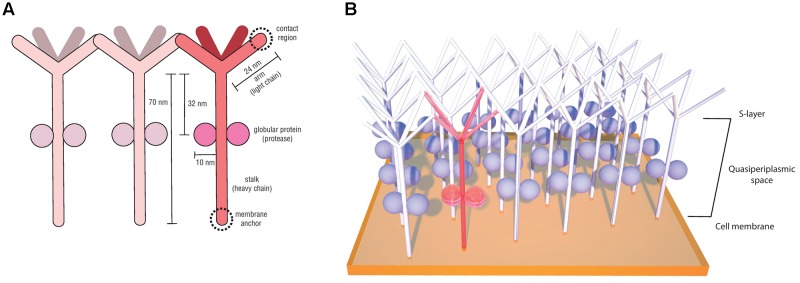
*Staphylothermus marinus* tetrabrachion structural components (based on the model proposed by Peters et al., 1995) **(A)** and schematic illustration of its interactions between morphological units on the cell surface (based on the model proposed by Mayr et al., 1996) **(B)**.

The first Desulfurococcaceae S-layer investigated in detail was from the species *Desulfurococcus mobilis* and was shown to exhibit units with an unusually low degree of order, with p4 symmetry and lattice constant of 18 nm (Wildhaber et al., 1987). Posterior structural analyses of the hyperthermophilic peptide-fermenting *Staphylothermus marinus* also revealed a p4 lattice symmetry with a similarly disordered surface meshwork (Peters et al., 1995). Meticulous imaging, biochemical and molecular studies (Peters et al., 1995, 1996; Mayr et al., 1996; Stetefeld et al., 2000) provided important insights regarding the singular morphology and structure of the *S. marinus* S-layer, where the subunits were described to resemble dandelion seed-heads. This morphological subunit (**Figure 5A**), denominated tetrabrachion, was shown to comprise a 70 nm stalk formed of four identical glycoproteins arranged in a coiled-coil domain ending in four “arms,” which are notorious for an extremely high amount of β-sheets. The 24 nm identical arms provide lateral connectivity by end-to-end contacts (**Figure 5B**) (Peters et al., 1995). A remarkable feature observed in the *S. marinus* S-layer was the presence of two globular proteins with proteolytic activity bound to the stalk at 32 nm from the arm branching point (Peters et al., 1995). This is the first and, to our knowledge, the only report of an archaeal S-layer component with enzymatic activity (Peters et al., 1996).

This protease is unusually resistant to heat and denaturation agents, especially when associated with the tetrabrachion, and for that reason it has been named STABLE (stalk-associated archaeal endo-) protease (Mayr et al., 1996). Sequence analyses indicated it to be a member of the subtilisin family and it was demonstrated to have broad substrate specificity. Based on its ability to cleave peptides in regions succeeding glutamate residues, it has been hypothesized that STABLE is a likely candidate for the cleavage of the tetrabrachion precursor during S-layer biosynthesis, given that the tetrabrachion heavy and light chains are part of a single gene product, with a cleavage site between Glu700 and Gly701 (Peters et al., 1996). Another suggested role for the STABLE protease is in providing the substrate necessary for *S. marinus* peptide fermentation metabolism (Fiala et al., 1986; Peters et al., 1996).

Crystallography analyses of the polypeptide fragment forming the coiled-coil domain of the *S. marinus* tetrabrachion stalk revealed yet another peculiar feature. While most coiled-coil motifs consist of two to five amphipathic α-helix containing heptad amino acid repeats which are intertwined into a left-handed super-helix, tetrabrachion stalk proteins were shown to form a coiled-coil structure comprising four α-helix with undecad repeats (11 amino acids) and a right-handed super-helix orientation (Stetefeld et al., 2000). This conformation results in four large cavities inside the tetrameric structure capable of binding large molecules, a feature that has been explored for biotechnological applications such as the development of drug delivery systems (Eriksson et al., 2009; McFarlane et al., 2009).

Another member of the Desulfurcoccacea family that had its cell envelope investigated is *Aeropyrum pernix*, which exhibits an S-layer with p4 lattice type with an open network of proteins and a relatively large periplasmatic space, features comparable to those observed in *D. mobilis* and *S. marinus* (Rachel, 2010). However, it is worth mentioning that although there are nine currently proposed genera within the family Desulfurococcacea (according to the 2015 edition of Bergey’s Manual of Systematics of Archaea and Bacteria), detailed descriptions of surface layers for most of these organisms are still unavailable.

The surface of *Pyrolobus fumarii*, an organism belonging to the family Pyrodictiaceae originally isolated from a hydrothermally heated black smoker wall at the Mid Atlantic Ridge, was also shown to consist of a crystal layer of tetrameric protein complexes arranged in a lattice with p4 symmetry, with center-to-center distances of 18.5 nm (Blöchl et al., 1997). Interestingly, all other organisms affiliated to the Pyrodictiaceae family described hitherto, including species of *Hyperthermus* and *Pyrodictium* genera, exhibit a surface layer with a hexagonal pattern (Stetter et al., 1983; Hegerl and Baumeister, 1988; Baumeister et al., 1990; Dürr et al., 1991; Rieger et al., 1995). However, three dimensional reconstructions have revealed that despite sharing the same lattice type, the S-layer from *Hyperthermus butylicus* is clearly distinct from those of *Pyrodictium* species, with differences in protein mass distribution, surface relief and larger spacing constants (Baumeister et al., 1990).

### Thermoproteales

This crenarchaeal group comprises rod-shaped thermophilic or hyperthermophilic microorganisms, growing either chemolithoautotrophically or by sulfur reduction of various organic substrates (Huber and Stetter, 2015b). Ultrastructure investigations of the species *Thermoproteus tenax* and *Thermoproteus neutrophilus* revealed S-layers with hexagonal lattices (p6) and center-to-center spacing values of around 30 nm, which is remarkably higher than reported for the hexagonal S-layers in Euryarchaeota (Messner et al., 1986). Despite the delicacy of the network, a notorious mechanical stiffness was reported for the S-layer of these organisms, suggesting a determinant or cell shape maintaining role (Wildhaber and Baumeister, 1987). Micrograph analyses revealed, as with other crenarchaeotes, a smooth exterior surface layer and a rough internal layer, with pillar-like protrusions interconnecting the thin layer to the plasmatic membrane, forming a 25 nm wide interspace (Wildhaber and Baumeister, 1987).

Very similar surface layer structures were later described for members of the genus *Pyrobaculum*, such as *P. islandicum* (Phipps et al., 1990), *P. organotrophum* (Phipps et al., 1991), *P. aerophilum* (Völkl et al., 1993) and *P. yellowstonensis* (Jay et al., 2015). As observed in organisms from the euryarchaeal order Thermococcales, the presence of a second layer apparently composed of dimers of single-domain subunits was reported in *P. organotrophum* (Phipps et al., 1991). This unique outer layer exhibited a simple architecture, with limited intersubunit connectivity. No defined orientation or distinguishable asymmetry between its inner and outer faces could be determined (Phipps et al., 1991). Recently, a similar outer sheath consisting of a single layer of small subunits, situated above the regularly organized hexagonal S-layer, was also observed in cells of *P. yellowstonensis* (Jay et al., 2015). However, the mechanism by which this outer layer interacts with the inner S-layer or its functions for these organisms remain elusive.

Unlike the Euryarchaeota, the crenarchaeotal S-layers are more heterogeneous in lattice type, center-to-center spacing and structural properties. However, there is a correlation between these properties among the different crenarchaeotal orders. Although the hexagonal S-layer lattice type is frequently associated with *Archaea*, in the Crenarchaeota phylum other lattice types are comparably common, with no clear predominance of any particular type. However, there seems to be a tendency when analyzing individual groups (**Figure 2**), with hexagonal S-layers being detected mostly in Thermoproteales. It is also worth pointing out that all archaeal S-layers composed of two different proteins were described in the crenarchaeotes. The role of glycosylation in the Crenarchaeota was investigated in Sulfolobales and seems to be correlated to cell viability in hot and acidic environments (Meyer and Albers, 2013).

## Other Archaeal Phyla

The Euryarchaeota and Crenarchaeota were the first archaeal phyla to be defined (Woese et al., 1990) and their members have received significant attention. However, with advances in molecular biology techniques, novel archaeal organisms that did not belong to either of those two phyla have been increasingly detected, leading to the proposal of a number of new taxonomic groups. It is worth mentioning that the majority of these new organisms were described mainly by culture-independent methods, limiting the information about their structural features, which naturally leads to less information regarding their cell envelope and consequently S-layer descriptions.

The Korarchaeota phylum was proposed based on phylogenetic analyses of recovered 16S rRNA gene sequences from a hot spring in the Yellowstone National Park, United States (Barns et al., 1996). In the following years, a composite genome of a member of this phylum was assembled from an enrichment culture and was given the name “*Candidatus* Korachaeum cryptofilum” (Elkins et al., 2008). In this study, electron microscopy images revealed a densely packed S-layer, which was thought to provide structural integrity for the cell in the presence of surfactants. Although details on the structure of the S-layer are still not defined, it has been suggested to be of hexagonal symmetry with a lattice constant of 14 nm (Rachel, 2010).

Another novel archaeal phylum where an S-layer has been detected is the Nanoarchaeota. This phylum was proposed after the discovery of very small 400 nm wide cells attached to another archaeon, *Ignicoccus hospitalis*, in samples recovered from a hydrothermal vent in Iceland (Huber et al., 2002). These cells were not able to grow without their host and were named as *Nanoarchaeum equitans.* Interestingly, the hexagonal S-layer has been reported to have an imperfect lattice (Junglas et al., 2008), with a periplasmatic space of 20 nm (Huber et al., 2003) and, based on the S-layer protein gene detection, an N-terminal signal peptide (Waters et al., 2003). The symbiotic nature of *N. equitans* and *I. hospitalis* has drawn attention from the scientific community, and because the *N. equitans* genome lacks genes for a number of essential components, it has been suggested that lipids and amino acids are transferred from its host (Waters et al., 2003). The cell surfaces of *I. hospitalis* and *N. equitans* can either be in complete contact or be close to one another (Junglas et al., 2008; Burghardt et al., 2009) which seems to be important in the compound exchange process between the two cells. In this context, questions concerning the role of the S-layer in *N. equitans* can be raised. However, there is no conclusive data on this matter, leaving it open to speculation.

The Thaumarchaeota phylum comprises the organisms which were previously classified as mesophilic crenarchaeotes, but have been shown to actually form a non-monophyletic sister group of the Crenarchaeota (Brochier-Armanet et al., 2008). Some of its members are notorious for being able to oxidize ammonia, a metabolic pathway previously thought to be exclusive to bacteria (Zhalnina et al., 2012). The first described thaumarchaeote was *Cenarchaeum symbiosum*, which grows symbiotically with a marine sponge (Preston et al., 1996). Although hypothetical proteins on its genome showed homology to known S-layer proteins, the occurrence of this cell envelope component has yet to be confirmed (Hallam et al., 2006). The ammonia oxidizing archaeon *Candidatus Nitrosopumilus maritimus* had an S-layer protein gene found in its genome and it has been suggested that the protein has a high number of reactive surface sites, which could increase affinity to ammonium ions (Gorman-Lewis et al., 2014). Another ammonium oxidizer, *Nitrososphaera viennensis*, had an S-layer detected on its surface. This organism was isolated from a garden soil sample in Vienna, Austria (Tourna et al., 2011) and it showcases a p3 lattice type on its S-layer with a center-to-center spacing of around 20 nm (Stieglmeier et al., 2014). This lattice type had been previously observed only in Sulfolobales and the lattice constant is also similar to that reported in this crenarchaeotic order (Veith et al., 2009). Based on its genome, it has been suggested that the protein also undergoes *N*-glycosylation processes, a characteristic that another archaeon of the same genus, *Candidatus* Nitrososphaera evergladensis, seems to share (Kerou et al., 2016). Although thaumarchaeotes are frequently detected in a variety of mesophilic environments through culture independent methods (Brochier-Armanet et al., 2008; Spang et al., 2010; Pester et al., 2012), information regarding their cell structure is limited compared to what is known in extremophiles. Thus, several aspects regarding archaeal biology in mesophilic environments remain to be explored.

## Concluding Remarks

There is an ongoing discussion on archaeal taxonomy, with a significant number of novel phyla being proposed in recent years, including Aigarchaeota, Geoarchaeota, Parvarchaeota, Aenigmarchaeota, Diapherotrites, Nanohaloarchaeota, Bathyarchaeota, Woesearchaeota, Pacearchaeota, Lokiarchaeota, and Thorarchaeota (Nunoura et al., 2011; Kozubal et al., 2013; Rinke et al., 2013; Meng et al., 2014; Castelle et al., 2015; Spang et al., 2015; Seitz et al., 2016). Although S-layers have been addressed, and sometimes thoroughly described in studies concerning organisms from the phyla discussed in this review, there is limited to no information on this topic regarding these above-mentioned novel phyla. As previously mentioned, the reason for this lack of literature has to do with the fact that most of these groups were proposed based on genome sequencing and culture independent methods, with no cultured isolates. There is a noticeably higher amount of data on archaeal S-layers from organisms belonging to the two initially described phyla, Euryarchaeota and Crenarchaeota, due to the isolation into pure culture of a sizable number of organisms from these groups. The difficulty of culturing new archaeal isolates has been frequently highlighted in the literature (Schleper et al., 2005; Vartoukian et al., 2010; Leigh et al., 2011) and this naturally hinders the amount of information that can be obtained on S-layers from these organisms.

On the basis of the archaeal S-layers described so far, the hexagonal lattice type seems predominant in absolute numbers. However, when the lattice type distribution among archaeal phyla is taken into consideration (**Figure 2**), it is evident that this lattice type is almost completely dominant in Euryarchaeota, especially in methanogens and halophiles, which have the highest number of cultured isolates. When analyzing the Crenarchaeota phylum separately, this lattice type can still be observed, but its dominance is not as clear as in Euryarchaeota and is mainly associated to the Thermoproteales order. Thus, it is difficult to draw a definite conclusion for *Archaea* as a whole, since the remaining archaeal phyla where an S-layer has been detected have too few described organisms for which this topic has been addressed. It is also worth pointing out that all archaeal S-layers composed of two proteins described hitherto have been detected exclusively in organisms from the Crenarchaeota phylum.

Although S-layers have properties that are common to all organisms described hitherto (e.g., self-assembly and lattice formation) the overall comparability between these gene and protein amino acid sequences is very low. As discussed in this review, sequence homology has been detected in specific groups but no global consensus has been established. A search for archaeal S-layer protein folding models in the Protein Data Bank (RCSB PDB) shows that there are only structural models for *Methanosarcina* spp. Thus, further studies are required to investigate if there are similarities in the overall folding of archaeal S-layer proteins, despite the low sequence homology. The role of S-layer glycosylation in thermophilic and halophilic archaea seems correlated to survival in extreme environments. In methanogens, a higher glycosylation density has also been detected in S-layer proteins from hyperthermophiles. Other poorly understood aspects of archaeal S-layer proteins include their interaction vesicles, viruses and their evolutionary history.

As previously discussed, S-layer coated vesicles in thermococci have been detected and because in some cases these vesicles carried DNA this has been suggested to be a mechanism for genetic transfer in high temperatures. However, further studies are necessary to assert the exact role S-layer proteins play in this process. Morphological changes in S-layers of *S. islandicus* and *S. shibatae* cells during virus egress have been reported (Daum et al., 2014; Quemin et al., 2016) but the specific interactions of viral components with archaeal S-layer proteins and S-layer structural changes involved during this process remain elusive. Interestingly, though there are few studies addressing this issue, similarities between some S-layer proteins and prokaryotic surface structures have been suggested. As previously mentioned, the *S. acidocaldarius* archaellum protein FlaF, which binds to the archaeal S-layer, exhibited a folding similar to the bacterial SbsB S-layer protein. Another curious similarity was reported between the archaeon “*Candidatus* Altiarchaeum hamiconexum” hami and known archaeal S-layer proteins (Perras et al., 2015). These hami are highly specialized nano-grappling hooks present on this organism’s surface. The hami protein subunits might be capable of self-assembly and have shown no similarity to known microbial surface structure proteins, such as those found in flagella, fimbriae and pili. However, their N-terminal region showed similarity to archaeal S-layers, suggesting a divergent evolution of these structures.

Finally, S-layer proteins are known to be suitable for different types of biotechnological applications and most *Archaea* described so far have an S-layer detected on the cell envelope. There have been very few studies exploring the potential of archaeal S-layer proteins, with the most noteworthy investigations applying the S-layer of *S. acidocaldarius* as template for gold (Au) nanoparticle production (Selenska-Pobell et al., 2011) and the *S. marinus* S-layer as a drug delivery system (Eriksson et al., 2009). Interestingly, both studies used crenarchaeotes as study models, with no reports in the literature using archaeal S-layers from other phyla for biotechnological purposes. Thus, archaeal S-layer proteins represent a large and unexplored field, both in basic and applied research, with new studies sure to advance our knowledge concerning this topic.

## Author Contributions

All authors listed have made a substantial, direct and intellectual contribution to the work, and approved it for publication.

## Conflict of Interest Statement

The authors declare that the research was conducted in the absence of any commercial or financial relationships that could be construed as a potential conflict of interest.
